# Prevalence and Risk Factors of Epilepsy Among the Adult Saudi Population: A Systematic Review

**DOI:** 10.7759/cureus.72294

**Published:** 2024-10-24

**Authors:** Thamer Alhumodi Alenazi, Ebraheem Lutfullah Khojah, Hassan Khafaji, Rahmah Majed Alsawad, Fatimah Mohammed Duleem Alqahtani, Farah Alghamdi, Faisal N Alhuwail, Alorayyidh Mohammed Mesal H, Yousef Salah Alharbi, Mohammed Alharbi

**Affiliations:** 1 Internal Medicine and Infectious Diseases, King Fahad Specialist Hospital, Tabuk, SAU; 2 Physical Therapy, Johns Hopkins Aramco Healthcare Center, Al-Ahsa, SAU; 3 Faculty of Medicine, King Abdulaziz University, Jeddah, SAU; 4 Medicine and Surgery, Dar Al Uloom University, Riyadh, SAU; 5 Surgery, Najran University, Najran, SAU; 6 Internal Medicine, King Faisal University, Al-Ahsa, SAU; 7 Medicine and Surgery, Shaqra University, Riyadh, SAU; 8 Medicine and Surgery, Jouf University, Sakaka, SAU; 9 General Practice, Al-Salam Hospital, Al Madinah, SAU; 10 College of Medicine, Qassim University, Unaizah, SAU

**Keywords:** adults, epilepsy, prevalence, public health, saudi arabia, systematic review

## Abstract

This study evaluates the prevalence of epilepsy within the Saudi population. A comprehensive computerized search of pertinent databases was carried out to find research that satisfied the requirements for inclusion. The search encompassed PubMed, SCOPUS, Science Direct, Cochrane Library, and Web of Science to find pertinent research. Our analysis included six studies with a total of 7408 Saudi participants. Females comprised the majority of the participants, totaling 6663 (89.9%). The prevalence of epilepsy among Saudi adults is underreported. Structural causes, such as cerebrovascular events and malignancies, were the most frequent etiologies, followed by genetic factors. A notable proportion of cases had an unknown cause. Gender-specific patterns were observed to show increased seizure severity in males whilst females showed age-specific vulnerability. This review emphasizes the complexity of epileptic conditions within the Saudi population, with diverse causes ranging from structural and genetic factors to strokes and head trauma. While males were more likely to experience severe seizures, females faced age-related vulnerability, pointing to the need for gender-sensitive treatment strategies. The lack of clear causes in nearly half of the cases further emphasizes the need for improved diagnostic approaches. However, the significant gap in prevalence studies within Saudi Arabia limits our understanding of the true burden of epilepsy in the region. Moving forward, comprehensive epidemiological studies focusing on prevalence and risk factors are crucial to inform public health strategies and improve outcomes for individuals living with epilepsy in Saudi Arabia.

## Introduction and background

Epilepsy is a neurological condition characterized by recurrent seizures. It is considered a global public health concern. While its prevalence varies across different populations, understanding its distribution and associated risk factors is crucial for effective prevention, diagnosis, and management [1]. 

Recent studies indicate fluctuations in the frequency of epilepsy in Saudi Arabia, with estimates ranging from 4 to 10 cases per 1,000 individuals. This discrepancy can be attributed to differences in diagnosis methodologies, population characteristics, and sociocultural factors across various regions of the Kingdom [2]. Despite its relatively high prevalence, epilepsy remains underdiagnosed and undertreated in many areas, particularly in rural and underserved populations. The stigma associated with epilepsy often exacerbates these challenges, leading to diminished quality of life and increased socioeconomic burdens for affected individuals and their families [3].

Saudi Arabia, a rapidly developing country with a diverse population, has seen significant changes in lifestyle and healthcare infrastructure in recent decades. These changes may have influenced the prevalence and risk factors of epilepsy. Adults in Saudi Arabia who have epilepsy are susceptible to comorbid factors associated with epilepsy [4]. These include genetic predispositions, traumatic brain injuries, cerebrovascular diseases, and infections such as neurocysticercosis. Additionally, lifestyle factors such as substance abuse, poor nutritional status, and inadequate healthcare access may further exacerbate the risk of developing this condition [5]. 

The perception and management of epilepsy are greatly influenced by cultural beliefs and practices from within Saudi society. Traditional attitudes toward mental health and neurological disorders often lead to delays in seeking medical assistance, which can worsen prognoses. Consequently, there is a pressing demand for culturally appropriate education initiatives that may close the gap between contemporary medical procedures and regional traditions [6].

The aims of this study are to investigate the prevalence of epilepsy in Saudi Arabia, identify the associated risk factors, and improve knowledge of the illness burden. This information can help with early diagnosis and treatment, guide the creation of focused preventative efforts, and ultimately improve the quality of life for those who have epilepsy. Understanding the prevalence and risk factors can aid in early diagnosis and intervention, ultimately improving patient outcomes. This systematic review may also contribute to the global body of knowledge on epilepsy, highlighting potential region-specific trends and disparities. Moreover, by identifying risk factors, the study can guide preventive strategies and enhance public health initiatives designed to reduce the burden of epilepsy.

## Review

Methods

This study followed the Preferred Reporting Items for Systematic Reviews and Meta-Analyses (PRISMA) standards while conducting a systematic review [7]. An electronic search was conducted to identify relevant English-language publications that examined the incidence and risk factors of epilepsy within the Saudi adult population. Databases including PubMed, Web of Science, SCOPUS, and Science Direct were all included in the search. The study included keywords linked to epilepsy and risk factors for epilepsy in our search approach. Two reviewers independently analyzed the results, selected eligible studies, retrieved data, and used assessment instruments to gauge the caliber of studies. 

Eligibility Criteria

The papers that explicitly address the prevalence and risk factors of epilepsy in the adult Saudi population were included in this systematic review. Studies that were considered eligible were English publications, including but not limited to original research articles, and studies that are cross-sectional, observational, cohort, and case-control in nature. Only studies that provided quantitative data on the prevalence of epilepsy or identify specific risk factors associated with the condition will be included. Conversely, the exclusion criteria disqualified studies that focus on populations outside of Saudi Arabia, studies conducted on children or adolescents, reviews, case reports, and editorials. Additionally, in order to maintain the emphasis and comprehensiveness of the evaluation, papers that did not provide pertinent data about the prevalence or risk factors of epilepsy were omitted.

Data Extraction

To ensure that the search results were accurate, Rayyan from Qatar Computing Research Institute (QCRI) was used [8]. Titles and abstracts from the search were evaluated for relevancy using the predetermined inclusion and exclusion criteria. Studies meeting these requirements were carefully examined by the research team. Disagreements were settled by consensus and debate. Using a predetermined extraction form, key research data, such as titles, authors, publication year, study location, participant demographics, gender distribution, and information on the epidemiology and risk factors of epilepsy in Saudi Arabia, were methodically documented. A neutral assessment instrument was developed to determine whether the included research had any possibility of bias.

Data Synthesis Strategy

A qualitative summary of the main components and research findings was provided via summary tables that were created utilizing data from pertinent studies. The best strategy for making use of the data from the included studies was identified once the systematic review's data collection was finished.

Risk of Bias Assessment

To evaluate the quality of the research, the Joanna Briggs Institute (JBI) critical assessment criteria for studies reporting prevalence data were used [9]. There are nine questions on this assessment form; a correct answer receives a score of 1 and a score of 0 is assigned to a negative, unclear, or irrelevant response. Scores were categorized into quality levels as follows: below 4 for low quality, between 5 and 7 for moderate quality, and above 8 for high quality. The articles' quality was evaluated by several researchers, and differences were settled through dialogue.

Results

Systematic Search Outcomes

**Figure 1:**
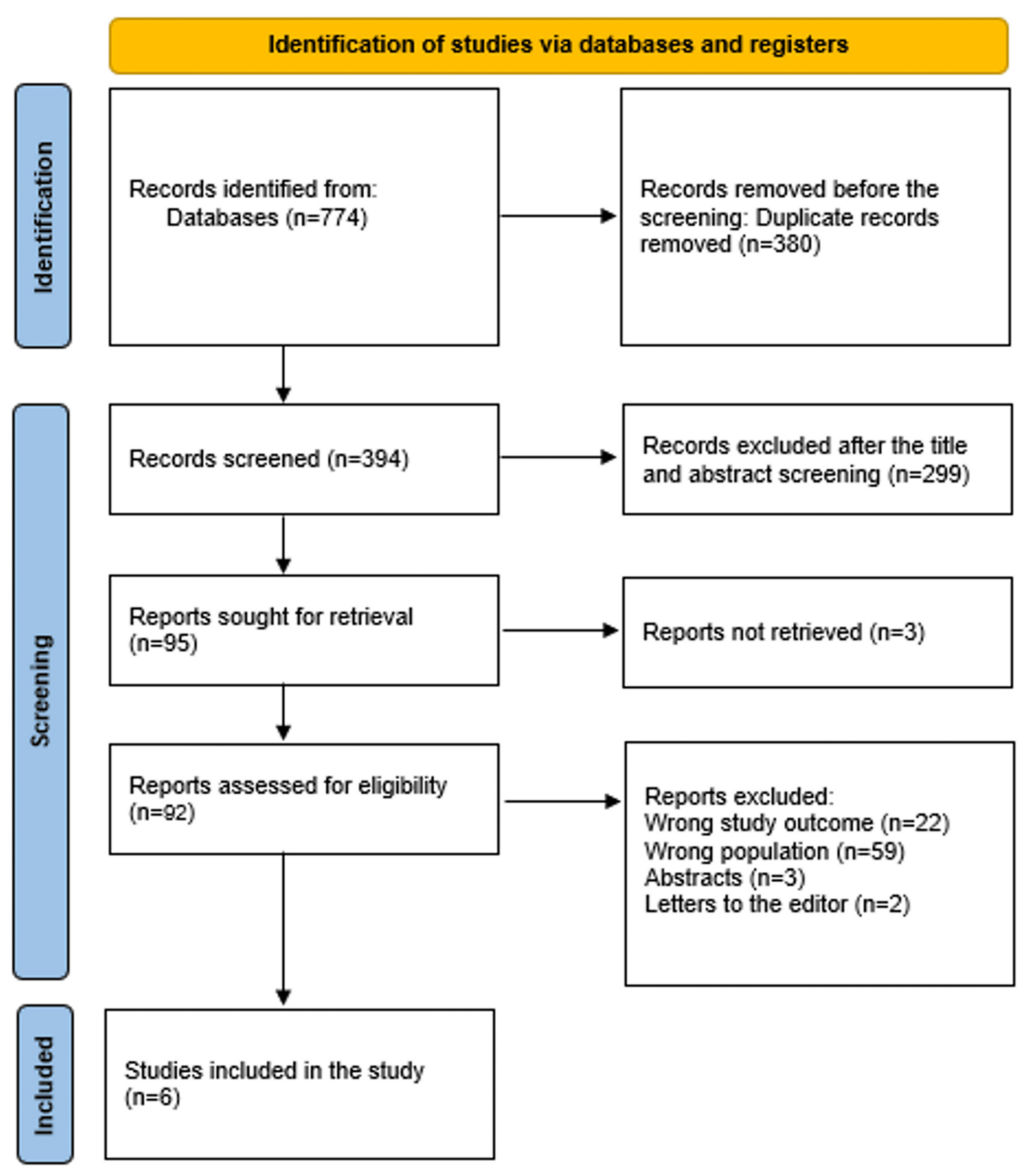
PRISMA diagram used to summarize the research choices. PRISMA: Preferred Reporting Items for Systematic Reviews and Meta-Analyses

A thorough search of 774 study papers yielded 380 duplicates that were disregarded. Two hundred and ninety-nine publications were discarded after 394 research' titles and abstracts were examined. Out of the 95 reports that were necessary, three were not found. Twenty-two papers were excluded because the study results were inaccurate, two were letters to the editor, and three were abstracts. Fifty-nine papers were disqualified for using the wrong demographic types. The qualifying requirements are met by the six research publications that comprise this systematic review. The process by which literature was selected is illustrated in Figure 1.

Sociodemographics and Clinical Parameters of the Involved Participants and Studies

Table 1 condenses the sociodemographic information found in the study publications. Our analysis included six studies with a total of 7408 Saudi participants. Females comprised the majority of the participants, totaling 6663 (89.9%) [10- 15]. The studies consisted of three retrospective cohort analyses [12,14,15], two cross-sectional studies [11,13], and one prospective cohort [10]. Geographically, two studies were conducted in Kobar [10,13] and one each in Jeddah [11], Al-Qassim [14], and Abha [15]. The first investigation was carried out in 2013 [15] and the earliest in 2023 [11,12].

**Table 1:** Sociodemographic characteristics of the included studies. NM: Not Mentioned

Study ID	Study design	Country	Participants (n)	Mean age	Females (%)
AlSheikh et al., 2020 [10]	Prospective cohort	Khobar	5872	28.8 ± 5.8	5872 (100%)
Makkawi et al., 2023 [11]	Cross-sectional	Jeddah	431	NM	213 (49.4%)
Faizo & Alrehaili, 2023 [12]	Retrospective cohort	Taif	150	NM	71 (47.3%)
Shahid et al., 2018 [13]	Cross-sectional	Khobar	184	35.4 ± 19.5	93 (50.5%)
Hamdy et al., 2014 [14]	Retrospective cohort	Al Qassim	341	24.5±17.2	189 (55.4%)
Babtain et al., 2013 [15]	Retrospective cohort	Abha	430	28	225 (52.3%)

Table 2 shows the clinical parameters. The prevalence of epilepsy among Saudi adults is very underreported as only one study [10] with 2.67% prevalence among pregnant women which is an underrepresentation population. In the general population, the most common causes of epilepsy were found to be structural in nature (42.9%), followed by genetic factors (7.2%), strokes (24.3%), and malignancies (23.8%) [11]. However, 47.6% of cases did not have a clearly identified cause. Male patients were observed to experience more severe seizures, while females appeared to be more affected at specific ages. Regional atrophy was shown to be the least frequent lesion in individuals with epilepsy, while white matter disease was found to be the most common in one study [12,15].

**Table 2:** Clinical parameters and outcomes of the comprised research. NM: Not Mentioned; JBI: Joanna Briggs Institute

Study ID	Population type	Epilepsy prevalence (%)	Risk factors	JBI
AlSheikh et al., 2020 [10]	Pregnant women	157 (2.67%)	NM	Moderate
Makkawi et al., 2023 [11]	General population	NM	The most prevalent cause was structural (42.9%), followed by genetic (7.2%). The most common structural causes were malignancies (23.8%) and strokes (24.3%). 47.6% of instances, meanwhile, had an ambiguous cause.	Moderate
Faizo & Alrehaili, 2023 [12]	General population	NM	Males had more severe seizures, whereas females were more afflicted at specific age groups. Furthermore, this study determined that white matter illness was the most typically exhibited lesion in epilepsy patients, while regional atrophy was the least prevalent type of lesion discovered.	Moderate
Shahid et al., 2018 [13]	General population	NM	Because epilepsy is still stigmatized by society, it is more common in men than in women, which explains why there aren't as many female epilepsy sufferers. The discrepancy between semiology and EEG data emphasizes how important it is to thoroughly describe the history because patients and observers both are prone to ignoring transient, localized symptoms.	Moderate
Hamdy et al., 2014 [14]	General population	NM	The most frequent causes of symptomatic epilepsy were head trauma and cerebrovascular accidents. The duration of the illness, EEG changes, and seizure control did not significantly correlate.	Moderate
Babtain et al., 2013 [15]	General population	NM	The most frequent causes of symptomatic epilepsy were head trauma and cerebrovascular accidents. The duration of the illness, EEG changes, and seizure control did not significantly correlate.	Low

Additionally, stigma and social perceptions were noted to influence the male epilepsy population, suggesting underreporting or delayed diagnoses in females. Furthermore, disparities in symptomology and EEG findings underscore the need for careful history-taking to capture localized symptoms that may be overlooked [13]. Cerebrovascular events and head trauma were identified as the most frequent causes of symptomatic epilepsy across studies, and no significant relationship was observed between seizure control, length of illness, and EEG alterations [14]. 

Discussion

The prevalence of epilepsy among Saudi adults is very underreported as there was only one study with a 2.67% prevalence among pregnant women which is an underrepresentation population [10]. In the general population, the most common causes of epilepsy were found to be structural in nature (42.9%), followed by genetic factors (7.2%), strokes (24.3%), and malignancies (23.8%) [11]. Male patients were observed to experience more severe seizures. Females appeared to be more affected at specific ages [12,15]. Additionally, stigma and social perceptions were noted to influence the male epilepsy population, suggesting underreporting or delayed diagnoses in females. Furthermore, disparities in symptomology and EEG findings underscore the need for careful history-taking to capture localized symptoms that may be overlooked [13]. Cerebrovascular events and head trauma were identified as the most frequent causes of symptomatic epilepsy across studies, and no significant relationship was observed between seizure control, length of illness, and EEG alterations [14]. 

This discrepancy has been explained by the observation that women are more likely to hide their epilepsy diagnosis than males are to sustain head traumas [16]. However, this finding contrasts with previous studies in the Arab community, which indicated that women had a greater frequency of epilepsy. Epidemiological studies in the Arab population further highlighted that this gender difference might stem from variations within the community [17]. Similarly, other global studies reported a female predominance [18]. It has been suggested that women may be more inclined to hide their condition due to the potential for social stigma and challenges with marriage prospects [18].

Furthermore, Garcia-Martin et al. found that the majority of seizures (75.5%) originated focally, compared to seizures with generalized onset (17.5%) [19]. In an investigation on adults with epilepsy (average age 31.5 years), Mac et al. found that the frequency of generalized seizures (78%) was higher than that of focal seizures (22%) [20]. In line with our findings, tonic-clonic seizures were prevalent among the generalized episodes. This observation is consistent with previous research conducted in Asian and African populations [21]. However, other investigations have identified a greater proportion of focal seizures [22]. This divergence has been attributed to challenges in recognizing early focal symptoms, often resulting in missed detection of the initial phase and only observing the generalized convulsions [23]. Additionally, some generalized seizures might have started with focal onset that evolved into bilateral tonic-clonic seizures, while cultural factors may have contributed to the underreporting of focal episodes [23].

There are numerous significant clinical implications for the review. The identification of structural and cerebrovascular causes as primary contributors to epilepsy suggests that early detection and intervention strategies targeting these risk factors could potentially reduce the burden of epilepsy in the Saudi population. Moreover, the gender-specific patterns observed suggest that healthcare providers should consider age and sex when diagnosing and treating epilepsy, as men and women may experience the condition differently. The significant proportion of cases with no clear cause underscores the need for advanced diagnostic tools and methods, such as improved neuroimaging techniques, to better identify underlying pathologies. Furthermore, the impact of social stigma on male patients highlights the importance of patient education and community outreach programs to raise awareness and reduce the stigma associated with epilepsy.

Strengths and Limitations

The evaluated papers provide important information about the prevalence and risk factors of epilepsy in Saudi Arabian society. One of the major strengths of this review lies in its inclusion of a range types of individuals within the population, including pregnant women and the general population as whole, providing a broad understanding of epilepsy's impact across different demographics. The research also highlights the diversity of epilepsy causes, such as structural, genetic, and cerebrovascular events, while acknowledging the challenges posed by cases with no clear cause. 

Despite these strengths, several limitations are evident in this body of research. A significant limitation is the lack of detailed Saudi prevalence studies on epilepsy, particularly in non-specific population groups. While some data on prevalence in pregnant women are provided, the absence of comprehensive prevalence data across the broader Saudi population limits the ability to fully assess the scope of epilepsy in the region. Another limitation is the variability in the methodologies and diagnostic criteria employed across studies, which may affect the comparability of findings. Additionally, some studies lacked detailed information on risk factors, particularly for pregnant women, leaving gaps in understanding the underlying causes and exacerbating factors for epilepsy in this demographic. The majority of research' reliance on cross-sectional data makes it more difficult to determine the causal links between known risk factors and the onset of epilepsy.

## Conclusions

This review emphasizes the complexity of epilepsy within the Saudi population, with diverse causes ranging from structural and genetic factors to strokes and head trauma. While males were more likely to experience severe seizures, females faced age-related vulnerability, pointing to the need for gender-sensitive treatment strategies. The lack of clear causes in nearly half of the cases further emphasizes the need for improved diagnostic approaches. However, the significant gap in prevalence studies within Saudi Arabia limits our understanding of the true burden of epilepsy in the region. Moving forward, comprehensive epidemiological studies focusing on prevalence and risk factors are crucial to inform public health strategies and improve outcomes for individuals living with epilepsy in Saudi Arabia.
